# Probing the Extent of Randomness in Protein Interaction Networks

**DOI:** 10.1371/journal.pcbi.1000114

**Published:** 2008-07-11

**Authors:** Joseph Ivanic, Anders Wallqvist, Jaques Reifman

**Affiliations:** Biotechnology HPC Software Applications Institute, Telemedicine and Advanced Technology Research Center, U.S. Army Medical Research and Materiel Command, Ft. Detrick, Maryland, United States of America; Columbia University, United States of America

## Abstract

Protein–protein interaction (PPI) networks are commonly explored for the identification of distinctive biological traits, such as pathways, modules, and functional motifs. In this respect, understanding the underlying network structure is vital to assess the significance of any discovered features. We recently demonstrated that PPI networks show degree-weighted behavior, whereby the probability of interaction between two proteins is generally proportional to the product of their numbers of interacting partners or degrees. It was surmised that degree-weighted behavior is a characteristic of randomness. We expand upon these findings by developing a random, degree-weighted, network model and show that eight PPI networks determined from single high-throughput (HT) experiments have global and local properties that are consistent with this model. The apparent random connectivity in HT PPI networks is counter-intuitive with respect to their observed degree distributions; however, we resolve this discrepancy by introducing a non-network-based model for the evolution of protein degrees or “binding affinities.” This mechanism is based on duplication and random mutation, for which the degree distribution converges to a steady state that is identical to one obtained by averaging over the eight HT PPI networks. The results imply that the degrees and connectivities incorporated in HT PPI networks are characteristic of unbiased interactions between proteins that have varying individual binding affinities. These findings corroborate the observation that curated and high-confidence PPI networks are distinct from HT PPI networks and not consistent with a random connectivity. These results provide an avenue to discern indiscriminate organizations in biological networks and suggest caution in the analysis of curated and high-confidence networks.

## Author Summary

A protein–protein interaction network represents the set of pair-wise associations that have been discerned between the constituent proteins of an organism. There are three main types of such networks: (i) those determined from a single high-throughput experiment; (ii) curated, where interactions are compiled from the literature; and (iii) high-confidence, which contain subsets of interactions from total sets that may comprise any from types (i) and (ii). The latter are deemed to better represent those interactions actually occurring in a cell. Through the use of graph-theoretic analyses and a random network connectivity model, we find that biological networks of type (i), determined from a single high-throughput experiment, contain random, indiscriminate, binding patterns. However, networks of type (ii) and type (iii) are not representative of the random model, suggesting that they contain biased influences upon the protein associations. These conclusions have been suspected for some time but are further clarified in this work. Our findings provide an avenue to detect unconstrained or completely random network structures and lend insights into the identification of preferentially connected networks resulting from the underlying biological processes or manual curation.

## Introduction

Protein interaction networks are key to the understanding and modeling of many biological processes. At the highest level, networks enable the conceptualization of the different physiological, biological, and chemical functions that typically occur in a cell. At the core of a network description lie the connections, or relationships, between the components present in a system, such as interactions, reactions, and modifications. Using high-throughput (HT) experimental techniques, large sets of component connections (blueprints) are now becoming available. Ultimately, for a cellular system, we desire the complete set of interactions between the constituent proteins (interactome) [1],[2]. The architectures of protein interaction networks, or their modes of assembly, are a consequence of how biological functions and processes have evolved and adapted over time. As such, it is imperative to analyze experimentally discovered biological networks from a number of perspectives, including mathematical.

Efforts to elucidate entire protein-protein interaction (PPI) networks for species have emerged in the forms of experimental HT technologies [3]–[6], large-scale curation [7], and predictive, or inferring, methodologies [8],[9]. To date, extensive PPI networks have been experimentally determined for a number of organisms, including *Saccharomyces cerevisiae* [10],[11], *Escherichia coli* [12],[13], *Helicobacter pylori* [14], *Drosophila melanogaster* [15], *Caenorhabditis elegans* [16], *Plasmodium falciparum* [17], *Campylobacter jejuni* [18], and *Homo sapiens* [7]. A number of efforts to compile and, in some cases, curate the data have emerged [7], [19]–[23], and the topological properties of these networks have been widely explored using a range of theoretical techniques [24]–[27]. A common feature of almost all biological networks is that their degree distributions roughly resemble a power law: *P*(*k*)∼*k* ^−*β*^, where *P*(*k*) is the probability of any component having *k* direct interactions (or degree *k*) and *β* is usually between one and three [28]–[30]. In fact, many real-world systems show power-law property distributions [31]. Whether or not PPI networks have a power-law degree distribution is under debate [32]; however, it is clear that in PPI networks proteins that have very low degrees (one or two) are prevalent, while there are very few proteins that have especially many interactions (tens to hundreds). A number of graph construction models are able to generate networks having power-law-type degree distributions, including those based on preferential attachment [33],[34], duplication [35]–[37], and hierarchical [38],[39] approaches. However, use of these models to reproduce a desired degree distribution, such as that observed for a particular experimentally determined PPI, is not straightforward. Therefore, it is difficult to ascertain precise levels of correlation between the models and the observed biological networks. In this respect, models that generate networks with given degree distributions are desirable. It is well known that Erdös-Rényi (ER) random graphs [40],[41] do not have power-law degree distributions, but variations of this model are able to generate random-type networks with desired degree distributions [42]–[45]. However, this type of graph has been reported to have topological properties that are generally different from PPI networks [46]–[48].

Many studies have aimed to discover biological insights from PPI networks. Avenues pursued to this end include the identification of salient protein clusters and functional modules [49]–[53]. Such biological entities usually occur as dense sub-graphs that are highly intraconnected but loosely connected to the remainder of the network. Consequently, procedures for identifying them have utilized graph-theoretical algorithms that analyze local and global topological network properties [50],[52],[53] and methods that include protein functional information [49]. Therefore, comprehension of the general organizational principles of PPI networks may serve to enhance the discernment and evaluation of biological modules.

In a previous study, we investigated the extent of preferential attachment, or degree-weighted (DW) behavior, in nine PPI networks [54]. It was demonstrated that, overwhelmingly, the probability of interaction of two proteins is proportional to the product of their degrees, i.e., *P_ij_*∝*k_i_k_j_*, where *k_i_* and *k_j_* are the degrees of proteins *i* and *j*, respectively. It was also surmised that degree-weighted behavior is a characteristic of randomness. Here, we expand upon these findings by utilizing a random network construction model that generates a DW network, while attempting to duplicate a given degree distribution. We show that networks generated with this DW model have topological properties that are consistent with PPI networks determined from single HT experiments. The results suggest that these experimental PPI networks exhibit random connectivity. However, the model fails to reproduce properties of curated and high-confidence PPI networks, suggesting that these are composed of multiple single-experiment modules, or, if not, that they exhibit constraints in their organizations.

It should be stressed that the actual probability of two proteins physically interacting, or binding, is unlikely to be random. Such an event is dependent on many factors, including the types of residues, or domains, on each protein, their conformations, and the presence of perturbing proteins. Here, we are investigating PPI networks of experimentally identified protein interactions from which the degrees of the proteins are given properties. The frequency, or likelihood, of interaction between two proteins of particular degrees is then a secondary quantifiable property. It is the latter characteristic that we find to be indicative of randomness. However, the degree distributions of PPI, and many real-world, networks are known to resemble power-law scaling and not Poissonian, or random, distributions. Hence, the non-random degree distributions seem anomalous with respect to the random connectivities. We reconcile this discrepancy by describing a model for the evolution of protein degrees that consists of sequential duplication and random mutation steps. This evolution process converges to a steady state for which the degree distribution is identical to one that has been calculated by averaging over eight HT PPI networks. The results suggest that our interpretation of random connectivities in PPI networks is consistent with a randomly influenced evolution of their degree distributions.

### Degree-Weighted Behavior in Protein–Protein Interaction Networks

**Figure 1:**
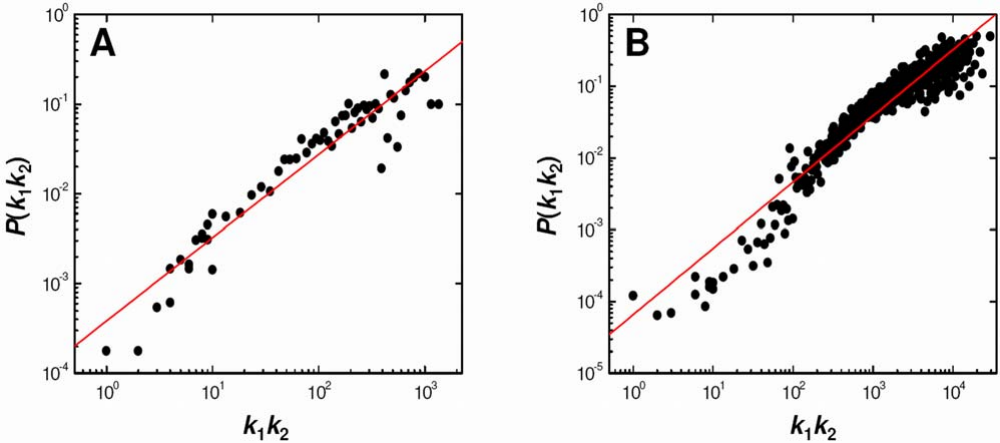
Evidence of Degree-Weighted Connectivity in Two PPI Networks. (A) *Helicobacter pylori* and (B) *Campylobacter jejuni*. For *k*_1_ *k*_2_>10, probabilities of interaction *P*(*k*_1_,*k*_2_) were ordered by *k*_1_ *k*_2_ and averaged in groups of 10.

Degree-weighted behavior, simply put, implies that the higher the degree of a node is the more likely it is to have an edge with any other node. Thus, the likelihood of an edge between two nodes is proportional to the product of their degrees, where the exact probability can be given by *P_ij_* = *γ*(*k_i_k_j_*)*^θ^*. In order to conserve the degree distribution, *θ* must equal one and *γ = E*/Σ*_i_*_<*j*_(*k_i_k_j_*), where *E* is the total number of edges in the network. It has been shown that these probabilities and constraints are overwhelmingly incorporated in PPI networks [54]. The only nodes that seem to show any deviation from DW behavior are those with very many connections, also known as hubs. Although the DW nature is less pronounced for these nodes, hub-hub interaction probabilities are still high. However, it is important to note that the level of noise in the hub-hub region varies from network to network. In fact, for some PPI networks, such as *P. falciparum* [17], the DW behavior is exemplary throughout [54]. Figure 1 shows the DW nature of two PPI networks not included in the previous study, *H. pylori* [14] and *C. jejuni* [18]. Note that we are plotting the dependence of the probability of interaction *P*(*k*_1_,*k*_2_) between two nodes of degrees *k*_1_ and *k*_2_ upon the product of their degrees *k*_1_ *k*_2_. These probabilities have been calculated by counting the total number of interactions occurring between all proteins of degree *k*_1_ and *k*_2_, and dividing this by the total number of all pairs of combinations that can be made.

The relation between DW behavior and the previously noted disassortive nature of biological networks [24],[55] is worth commenting on. Disassortiveness implies that high-degree nodes prefer to connect to low-degree nodes. Seemingly in contrast, DW behavior implies that if a node is given a choice of two potential interacting partners, it will more often connect to the one of higher degree. However, in a typical PPI network the number of high-degree nodes is magnitudes less than the number of low-degree nodes. Therefore, while a high-degree node may make *many* connections to low-degree nodes, and appear disassortive, the observation that it makes *any* connections with other high-degree nodes is significant. We have demonstrated that in PPI networks, high-degree nodes are almost always within one or two steps from each other [54]. This characteristic is exemplified in Video S1, which contains a three-dimensional animation of the HT *E. coli* PPI network determined by Arifuzzaman et al. [13].

### Previous Degree-Weighted Network Models

A recently reported model, denoted “STICKY,” has been likened to PPI networks [47]. This model uses the probability of interaction between two nodes to be proportional to the product of their input weights, which are the experimentally observed degrees [47]. By allowing for self-interactions and normalizing for the total number of edges, *E*, the probability of an edge between nodes *i* and *j* is given by *P_ij_* = (*k_i_k_j_*)/(4*E*), where the factor four arises due to double looping. The STICKY procedure enumerates through all pairs of nodes twice (once for *i = j*) and assigns an edge if a uniformly generated random number is larger than *P_ij_*. However, it was not reported that this procedure produces degree distributions that are different from the experimental input degree distribution. We observe that due to the nature of the edge-sampling procedure, the eventual degree of a chosen node can be modeled by a probability curve that is Poissonian about its expected, or input, degree. In other words, if a node has input degree *k*, then after many realizations of the STICKY procedure, i.e., multiple complete network constructions, the set of observed degrees for that node will follow
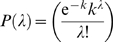
(1)where *P*(*λ*) is the fraction of networks in which the node has a degree *λ*. For nodes of low degree (one or two), their Poissonian distributions (Equation 1) are substantially skewed towards *λ* = 0. This means, for example, that in a typical STICKY network construction, 36.8% of nodes of input degree one will remain degree one, while 36.8% will become degree zero. Our computational simulations of the STICKY procedure consistently generate observed degrees in line with those predicted from Equation 1. Generally, in PPI (and most real-world) networks, nodes of degree one are most prevalent. Therefore, a model that strictly preserves their degrees, rather than letting many become zero, is desired for a fair comparison between model and experiment. If two networks have varying degree distributions, it is likely that their underlying architectures are different, regardless of any similarities in other global topological properties.

## Materials and Methods

### Protein–Protein Interaction Networks

A total of 12 PPI networks were included in the study and these were partitioned into two groups. The references immediately following the network species/labels represent the direct sources. These encompass original publications [11]–[18],[20], the Database of Interacting Proteins (DIP) [19], and the Human Protein Reference Database [7]. The first group contains eight PPI networks that have each been determined from an individual HT experiment using either yeast two-hybrid (Y2H) or tandem affinity purification (TAP) methodology: *C. jejuni* [18], *E. coli* (*HT1*) [12], *E. coli* (*HT2*) [13], *C. elegans* (*Y2H*) [16], *S. cerevisiae* (*Y2H*) [11], *H. pylori* [14], *P. falciparum* [17], and *D. melanogaster* [15]. Only the two PPI networks of *E. coli* were evaluated using TAP technology, all others were determined using Y2H methodology.

The second group contains four PPI networks that are either (i) merged experimental datasets: *H. sapiens* [7] and *S. cerevisiae* (*DIP*) [19]; (ii) inferred high-confidence from multiple datasets: *S. cerevisiae* (*CORE*) [19],[20]; or (iii) high-confidence from an individual experimental study: *C. elegans* (*CORE*) [16]. The number of proteins and interactions in each network is given in Table 1.

**Table 1:** Properties of 12 PPI Networks and Their Corresponding DCDW Networks.

				〈*C*〉		〈*L*〉	
Network	Ref	Number of proteins	Number of interactions	PPI	DCDW^a^	PPI	DCDW^a^
*C. jejuni*	[18]	1331	11664	0.095	0.095	2.91	2.85
*E. coli* (*HT1*)	[12]	1289	5420	0.083	0.089	3.60	3.29
(*HT2*)	[13]	3047	11477	0.064	0.085	3.37	3.27
*C. elegans* (*Y2H*)	[16]	2624	3967	0.020	0.017	4.81	4.36
*S. cerevisiae* (*Y2H*)	[11]	3277	4393	0.018	0.025	4.88	4.50
*H. pylori*	[14]	724	1403	0.015	0.025	4.15	4.06
*P. falciparum*	[17]	1304	2745	0.014	0.012	4.26	4.26
*D. melanogaster*	[15]	6986	20243	0.009	0.006	4.46	4.32
*S. cerevisiae* (*CORE*)	[19],[20]	2449	5579	0.207	0.007	5.21	4.49
*H. sapiens*	[7]	9263	34564	0.102	0.011	4.28	3.98
*S. cerevisiae* (*DIP*)	[19]	4617	16311	0.099	0.014	4.12	3.91
*C. elegans* (*CORE*)	[16]	727	814	0.030	0.010	5.40	4.83

a Averaged over 100 realizations.

### A Degree-Conserving Degree-Weighted Model

Given a set of nodes and their degrees, we consider a DW model that constructs a corresponding network while conserving the node degrees. Rather than considering every unique pair of nodes once (in any order) and (for each pair) generating a uniform random number to test whether an edge is assigned between them, as in the ER random [40],[41] and STICKY [47] construction procedures, we consider each unassigned edge once, for a given node, and use uniform random numbers to determine which other node it will connect to. This principle is not unlike that of previous preferential attachment models [33],[34] except that here it is used to generate networks for which each node has a specified degree, instead of growing, or evolving, them from seeds [56]. In the degree-conserving degree-weighted (DCDW) model, each node is considered once, in a random order, and a set number of edges are placed between itself and a DW random selection of the rest of the nodes. For each considered node, the remaining (potentially interacting) nodes are sampled for by using their input degrees as probability weights. However, none of the nodes are allowed to have more interactions than their given, or input, degrees. For an input degree sequence, which defines the desired degree *k_i_* of each node *i*, the DCDW model is defined by the following procedure:

1. Enumerate all nodes once in random order For the randomly-selected node *i*, *k_i_* is the input degree and *m_i_* is the number of edges previously connected to it
2. Enumerate the termination of (*k_i_*−*m_i_*) edges originating from node *i*
3.
  1. For each edge, choose the terminus node, *ℓ*, at random from the remaining nodes {*j*} using their input degrees, *k_j_*, as probability weights.
  2. If an edge already exists between nodes *i* and *ℓ* repeat preceding step (i).
  3. If connecting nodes *i* and *ℓ* will cause node *ℓ* to have more edges than its input degree, *k_ℓ_*, disregard the edge and repeat preceding step (i).

This model generates a DW network, such that all nodes have the desired degree without including self interactions. In our computations, there are very rare instances when step (2) is unable to complete. In this case, we retain the edges that have been set and skip to the next node, which is determined at random. However, such an occurrence is extremely rare and has no real impact on the final degree distribution.

The DCDW model appears similar in style to the random network model of Newman, Strogatz, and Watts [44], which generates a random graph with a given degree distribution. In this latter model, each node is assigned a number of stubs equal to the desired degree of the node. These stubs represent incomplete edges that emerge from their respective nodes. The random network is then constructed by choosing pairs of stubs (on different nodes) at random and placing edges between them. Thus, it can be construed that the probability weight of a node, at any time, is proportional to the number of unconnected stubs. Therefore, the probability weight of each node will slowly diminish as its stubs are used up. In contrast, the DCDW model uses constant probability weights for the nodes (proportional to their input degree) throughout the network construction procedure. As a result, the DCDW method is more likely to generate a true DW graph in which the probability of an edge between two nodes is proportional to the numerical product of their eventual degrees. In a way, the DCDW model can be thought of as being a mode of implementation of the method proposed by Newman, Strogatz, and Watts [44], although strictly speaking, the DCDW method generates a random DW graph.

**Figure 2:**
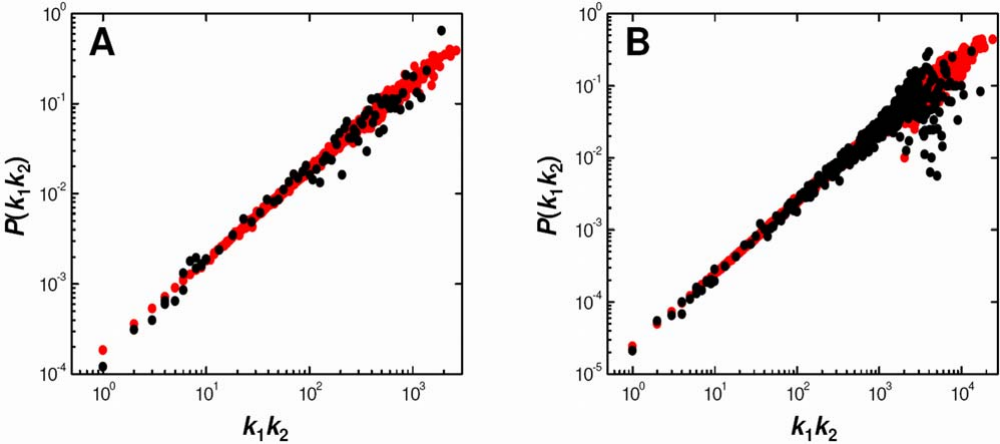
Degree-Weighted Connectivity in Two PPI Networks and Their DCDW Equivalents. (A) *Plasmodium falciparum* and (B) *Drosophila melanogaster*. Points in black correspond to the PPI networks and points in red correspond to their DCDW equivalents. For the PPI networks, probabilities of interaction *P*(*k*_1_,*k*_2_) were ordered by *k*_1_ *k*_2_, and values for *k*_1_ *k*_2_>10 were averaged in groups of 10. For the DCDW networks, probabilities of interaction were averaged over 100 realizations and all values are shown.

We demonstrate, using two examples, that the DCDW model effectively generates true DW graphs. Figure 2 illustrates the DW nature of the *P. falciparum* and the *D. melanogaster* PPI networks (black points) together with their equivalent (same input degree distributions) DCDW networks averaged over 100 constructions (red points). Two elements are evidenced from the plots: (i) The DCDW model, as expected, generates DW networks, and (ii) the PPI networks exhibit very similar DW behavior to their DCDW equivalents. We observe similar plots for all PPI networks studied here. The network of *D. melanogaster* shows slightly more noise than its DCDW counterpart in the hub-hub region; however, this is expected from previous observations [54]. It is also important to note that because PPI networks are generally construed from a single measurement of the interactions, they are prone to more noise.

## Results

### Connection between Degree-Weighted Behavior and Randomness

We recently illustrated, through simulations, that Erdös-Rényi (ER) random graphs [40],[41] show near-perfect DW behavior [54]. It can be analytically shown that any random graph will show DW connectivity. Details are provided in Text S1. We conclusively demonstrate this by constructing an ER random graph equivalent of the *P. falciparum* network (where the probability of any edge is determined from the number of nodes and edges in the *P. falciparum* PPI network) 10^4^ times, and for each construction we use the resultant degree distribution as input for the generation of a DCDW network. For each pair of networks, ER and DCDW, in each simulation, we calculate the number of assigned edges, average clustering coefficients (〈*C*〉), average shortest path lengths (〈*L*〉), and diameters (largest shortest path length) and then we average these properties over the 10^4^ simulations. The clustering coefficient of a node *i* is defined as the fraction of possible edges between neighbors that are present, where a neighbor of node *i* is any other node that shares an edge with it [57]. The average clustering coefficient of a network, 〈*C*〉, is determined by averaging the clustering coefficients of all nodes, where nodes of degree one are defined here to have a clustering coefficient of zero. The shortest path length between two nodes is the minimum number of steps (or edges) that must be traversed in order to go from one to the other. The average shortest path length of a network, 〈*L*〉, is the average of all shortest path lengths that are not undefined. The results are given in Table 2. It is found that all of the aforementioned properties are essentially identical for the ER random and the DCDW networks. Thus, it appears that, *given an input degree distribution indicative of an ER random network, the DCDW model will regenerate the ER random connectivity*. The methods of construction for the two networks are very different; the ER random model uses a constant probability for the assignment of an edge between any two nodes, whereas the DCDW model scales the probability of an edge between two nodes with the product of their degrees.

**Table 2:** Properties of an Erdös-Rényi (ER) Random Graph and the Corresponding DCDW Model^a^.

Network	Number of nodes	Number of assigned edges	〈*C*〉 (×10^−3^)	〈*L*〉	Diameter
ER	1304	2745.25	2.96	5.12	10.75
DCDW	1304	2744.94	3.10	5.12	10.78

a Results for both networks averaged over 10^4^ realizations.

**Figure 3:**
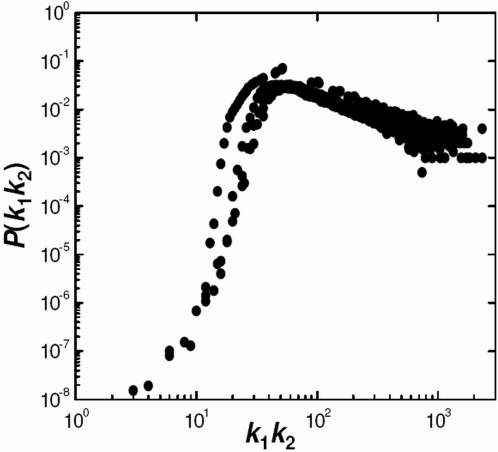
Example of Non-Degree-Weighted Behavior in Networks Generated from a Non-Random Model. The DCDW model was modified to eliminate both random enumeration of nodes and degree-weighted sampling for interacting partners. The degree distribution corresponding to the protein–protein interaction network of *Plasmodium falciparum* was used as input for the modified model.

We must conclude from the above findings that *random networks are inherently DW* and, conversely, that *DW behavior implies randomness in the connectivities*. A question is immediately realized: is it possible for a graph to *not* show uniform DW behavior? If our conclusion that DW behavior and randomness are synonymous is true, then removal of random and DW elements from a network construction process might yield networks that are not uniformly DW in their connectivities. We illustrate such an instance by modifying the DCDW procedure described above in two ways: firstly, in step (1), rather than enumerating all nodes once in random order, we enumerate all nodes *i* in order of decreasing degree; and secondly, in step (2), rather than weighting each of the possible interacting nodes (for node *i*) by their input degree, we weight them by the inverse of their input degree, i.e., *P*(*i*−*j*)∝1/*k_j_*. We use the degree distribution of the *P. falciparum* network as input and average probabilities of interaction over 1000 network constructions. Figure 3 illustrates the resulting dependence of the probability of interaction *P*(*k*_1_,*k*_2_) between two nodes of degrees *k*_1_ and *k*_2_ upon the product of their degrees *k*_1_ *k*_2_. Probabilities that are exactly zero, e.g., *P*(1,1) = *P*(2,1) = 0, are not shown. It is clear that this modified network construction procedure generates networks for which the connectivities deviate significantly from uniform DW behavior. Therefore, we can surmise that if a network does not show uniform DW behavior, it likely has been generated with some limiting condition(s).

### Comparison of the Degree-Conserving Degree-Weighted Model with Protein–Protein Interaction Networks: Global Properties

It has been previously established that PPI networks have a DW nature [54]. We have seen above that the DCDW model generates networks that are DW, while conserving a desired degree distribution. It was also demonstrated that random networks are intrinsically DW and that the DCDW model produces random networks for a given degree distribution. It remains to discover whether the DCDW model generates graphs that share topological characteristics with PPI networks. As a first step, we compute global properties of PPI networks and their equivalent DCDW networks (same input degree distributions). Table 1 provides the average clustering coefficients, 〈*C*〉, and the average shortest path lengths, 〈*L*〉, for the 12 PPI networks (undirected with no self interactions) and their DCDW equivalents, where values for each of the latter are averaged over 100 realizations. As described previously, the PPI networks in Table 1 are partitioned into two groups. The top eight are each taken from an individual HT experiment, whereas the bottom four are either (i) merged experimental datasets (*H. sapiens* and *S. cerevisiae* (*DIP*)), (ii) inferred high-confidence from multiple datasets (*S. cerevisiae* (*CORE*)), or (iii) high-confidence from an individual experimental study (*C. elegans* (*CORE*)). The PPI networks in each group have been arranged by decreasing average clustering coefficient.

We find that, for the first group, or the top eight networks in Table 1, average clustering coefficients of the experimental networks and the DCDW model are in excellent agreement. In fact, the DCDW model has values within 0.003 of the experimental for four systems (*C. jejuni*, *C. elegans* (*Y2H*), *P. falciparum*, and *D. melanogaster*) and within 0.007 for two (*E. coli* (*HT1*) and *S. cerevisiae* (*Y2H*)). The largest discrepancy of 0.021 is observed for the *E. coli* (*HT2*) network; however, the DCDW-determined value of 0.085 is still quite close to the experimental value of 0.064. The results clearly indicate that the DCDW model is accurately simulating the global clustering in PPI networks determined from individual HT experiments. In terms of the average shortest path lengths, the DCDW model predicts values within 0.14 for five systems in the first group (*C. jejuni*, *E. coli* (*HT2*), *H. pylori*, *P. falciparum*, and *D. melanogaster*). For the remaining three systems, the DCDW model predicts average path lengths that are somewhat smaller than the experimentally observed values. One reason for this is that in some PPI networks the DW behavior tends to level off in the hub-hub interaction region. As the DCDW model uses DW behavior throughout, it may generate slightly more hub-hub connections than are actually present. In such a case, and if many of the shortest path lengths utilize the hub proteins, one might expect the DCDW model to produce networks having slightly shorter path lengths than the actual PPI networks. However, this is observed for only three out of the eight PPI networks in the first group. Overall, the DCDW model predicts average clustering coefficients and shortest path lengths that are in good agreement with those of PPI networks determined from individual experiments. Furthermore, the orderings of the predicted and experimental values of each topological property are almost identical. These results lend further support to the presumption that DW behavior is intrinsic to these networks.

For the second group of PPI networks, which are either merged from multiple experimental datasets, high-confidence, or a combination of both, the DCDW-predicted clustering coefficients are far smaller than the actual values. In fact, for three systems (*S. cerevisiae* (*CORE*), *H. sapiens*, and *S. cerevisiae* (*DIP*)), the DCDW predictions are about a magnitude smaller. Average path lengths determined from the DCDW model are also consistently smaller than the true values, by over 0.50 in two instances (*S. cerevisiae* (*CORE*) and *C. elegans* (*CORE*)). The discrepancy is slightly less for the network of *S. cerevisiae* (*DIP*) (0.21). However, it must be concluded that the DCDW model fails to reproduce global properties of the PPI networks in this second group.

Given the success of the DCDW model with regard to the first group of PPI networks, the subsequent failure of this model when applied to the networks of the second group is initially unexpected. The networks in the first group are different from those of the second group in that each of the former are derived from a single experiment. Three of the networks in the second group (*S. cerevisiae* (*CORE*), *H. sapiens*, and *S. cerevisiae* (*DIP*)) have been assembled by the merging of multiple datasets. If the numbers of common proteins, or overlapping nodes, between pairs of datasets comprising a merged set are small, then, in effect, this merged set incorporates somewhat separated PPI sub-networks. For such a case, one would not expect the DCDW model to perform adequately because it does not incorporate constraints about which nodes are able to interact. Similar reasoning, in terms of artificially introducing selective connectivity, may be used to explain why the DCDW model cannot reproduce properties of the two high-confidence PPI networks *S. cerevisiae* (*CORE*) and *C. elegans* (*CORE*). Examination of the average shortest path lengths for the PPI networks in the second group indicate that they are much larger than for networks in the first group that have a similar clustering coefficient. This observation seems to corroborate the notion of multiple PPI sub-network contents and/or constrained connectivity.

### Comparison of the Degree-Conserving Degree-Weighted Model with Protein–Protein Interaction Networks: Clustering and Path Length Profiles

It is clear that the DCDW model generates graphs that have similar global properties to PPI networks determined from a single HT experiment. Given this affinity, it is worth comparing their inner architectures further. We accomplish this by examining the behavior of node degree versus clustering coefficient and average shortest path length. No additional analyses are performed upon the networks of the second group given that their global properties, in particular the clustering coefficients, are substantially different from those of the DCDW model.

**Figure 4:**
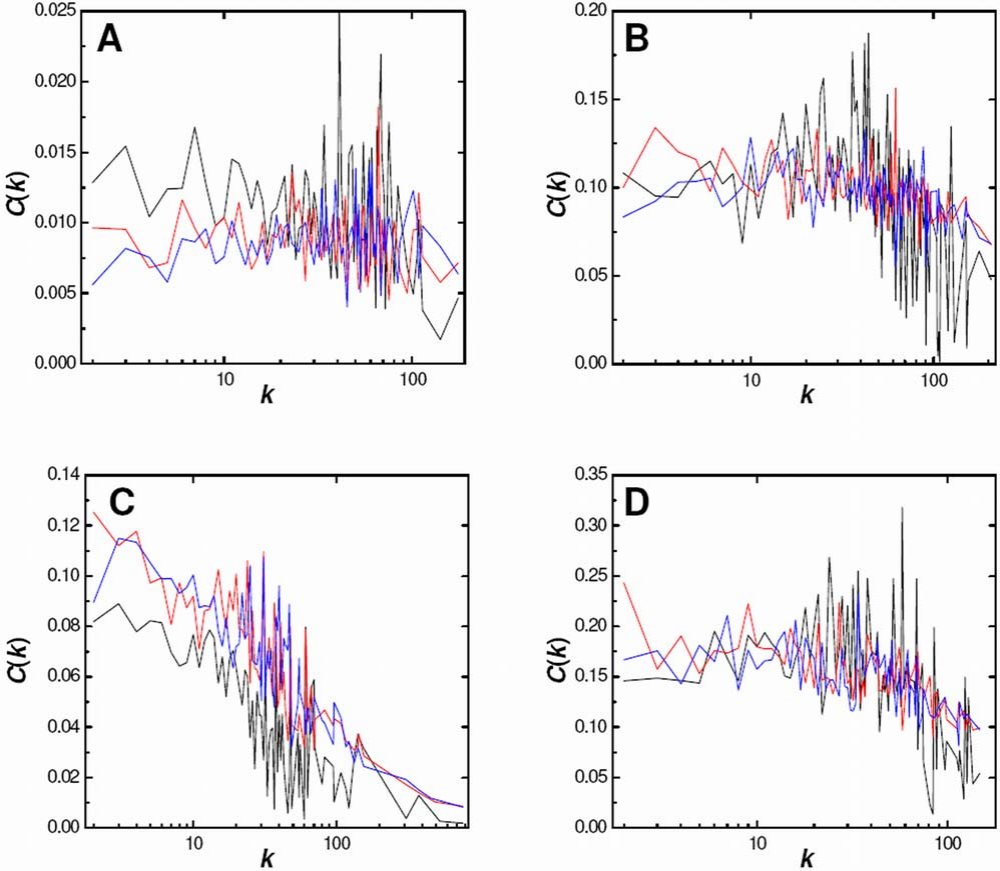
Dependence of Clustering Coefficient upon Node Degree for Four PPI Networks and their DCDW Equivalents. (A) *Drosophila melanogaster*, (B) *Campylobacter jejuni*, (C) *Escherichia coli* (*HT2*), and (D) *Escherichia coli* (*HT1*). Clustering profiles for the PPI networks (black) and the corresponding tenth (blue) and fiftieth (red) realizations of the DCDW model.

Clustering coefficient profiles are determined by evaluating the average clustering coefficient for nodes having the same degree. In this way, we elucidate the behavior of degree versus clustering coefficient. This type of analysis has been reported previously for *S. cerevisiae* PPI networks [46],[49] and metabolic networks [39]. Clustering profiles for the four largest PPI networks of the first group are shown as solid black lines in Figure 4. It is immediately apparent from the plots that the clustering coefficients do not vary smoothly with degree. Fluctuations start out small, relatively, in the low-degree regions but become wild as the degree is increased. However, there appears to be an overall trend in that clustering coefficients seem to decrease, somewhat, as the degree becomes large. This trend has been noted previously [39],[46],[49], and, although masked by large deviations, is most apparent here for the *E. coli* (*HT2*) network (Figure 4C) and least pronounced for the PPI network of *D. melanogaster* (Figure 4A). Clustering profiles are also shown for the corresponding DCDW model for the tenth (blue) and fiftieth (red) network realizations. We find that profiles for the two realizations are similar although it is clear that the DCDW model allows for some variation. The DCDW model, to some extent, reproduces the wild fluctuations of the experimental data. Correlation coefficients between profiles for the two DCDW realizations can be unexpectedly low, 0.05 and 0.15 for *D. melanogaster* (Figure 4A) and *C. jejuni* (Figure 4B), respectively, or considerable, 0.76 and 0.45 for *E. coli* (*HT2*) (Figure 4C) and *E. coli* (*HT1*) (Figure 4D), respectively. These correlations suggest that clustering coefficients are less constrained in the degree distributions of *D. melanogaster* and *C. jejuni*, while more limited for the distributions of both *E. coli* networks. These variabilities are reflected in the correlation coefficients between the experimental and the two DCDW profiles, which are lowest for *D. melanogaster*, −0.04 and 0.18, and highest for *E. coli* (*HT2*), 0.59 and 0.75. However, the large fluctuations in all profiles make adequate comparisons difficult. Nonetheless, no striking differences are observed and the overall DCDW profiles tend to follow those of the PPI networks, especially for the *E. coli* (*HT2*) network. Therefore, we can conclude that the DCDW model is reproducing features of the intrinsic clustering for these PPI networks. Analogous plots for the remaining four PPI networks of the first group are provided in Figure S1 and similar conclusions can be drawn from them.

**Figure 5:**
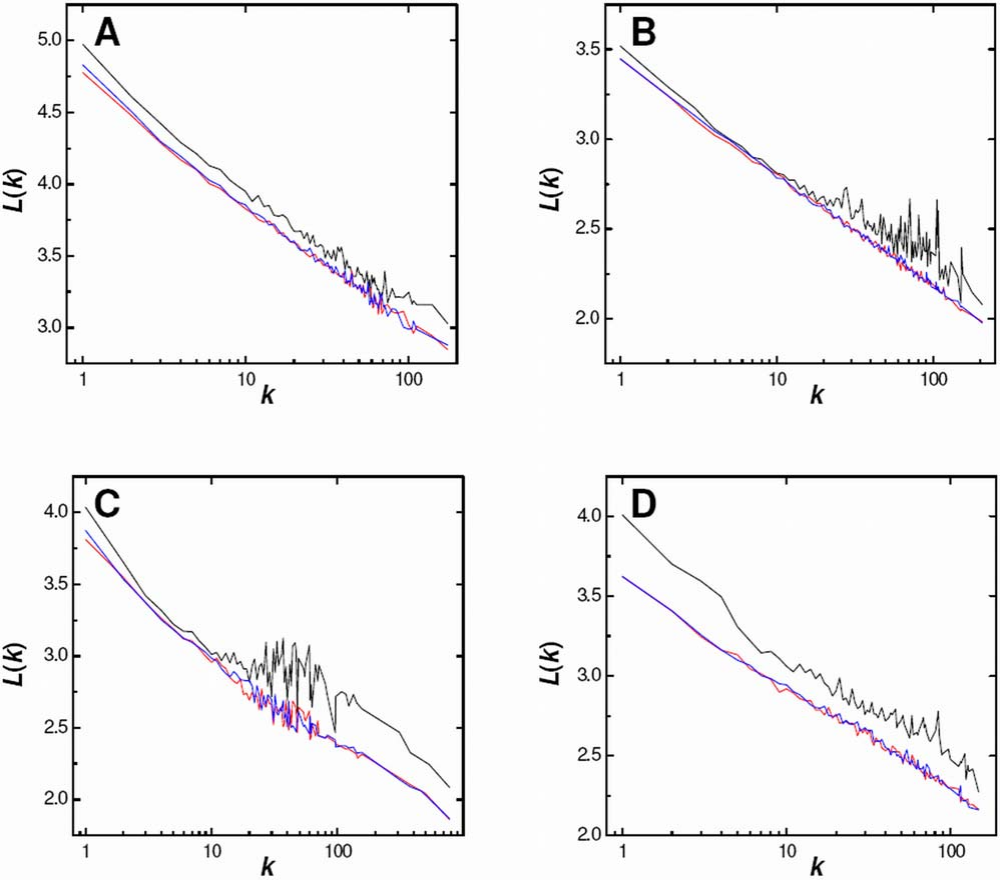
Dependence of Path Length upon Node Degree for Four PPI Networks and their DCDW Equivalents. (A) *Drosophila melanogaster*, (B) *Campylobacter jejuni*, (C) *Escherichia coli* (*HT2*), and (D) *Escherichia coli* (*HT1*). Path length profiles for the PPI networks (black) and the corresponding tenth (blue) and fiftieth (red) realizations of the DCDW model.

The average path length for a node, also known as closeness, is evaluated as the average number of steps connecting it to all other nodes. Path length profiles are determined by averaging closeness over nodes having the same degree. The dependence of closeness upon the degree has been studied previously for three PPI networks [58]. Path length profiles for the four largest PPI networks of the first group are shown as solid black lines in Figure 5. It is clear that the average path length consistently, and smoothly, varies inversely with the degree, indicating that nodes of higher degree are more central in the networks. This observation has been noted previously [54],[58]. Path length profiles are also shown for the corresponding DCDW model for the tenth (blue) and fiftieth (red) network realizations. It is evident that the DCDW model is reproducing the path length features of the PPI networks. While values for the DCDW model are consistently less than those of the corresponding PPI networks, the lines run almost parallel. Near-perfect agreement is observed for the *C. jejuni* network (Figure 5B) and the greatest variation is seen for the *E. coli* (*HT2*) network (Figure 5C). Not only does the DCDW model have a very similar path length dependence upon the degree as the PPI networks, it also incorporates the characteristic increased fluctuations noted at higher degree. Similar conclusions are drawn from corresponding path length profiles of the other four PPI networks in the first group and these are shown in Figure S2.

The affinities in clustering and path length profiles between the DCDW model and the PPI networks of the first group corroborate the findings of the previous section, in which similar corresponding global properties were observed. The DCDW model consistently produces networks that have similar global clustering coefficients to the corresponding PPI networks and the model also reproduces important features of the clustering profiles. Although global average path lengths can be smaller for the DCDW model, the generated path length profiles almost parallel those of the PPI networks. Therefore, we must conclude that the DCDW model is a plausible representation of PPI networks determined from a single HT experiment. As was demonstrated earlier, the DCDW model is representative of randomness and so we must conclude that these PPI networks incorporate a substantial random element. However, it is clear that the PPI networks of the second group, which are merged, curated and/or high-confidence datasets, are not well described by the DCDW model. The DCDW model only incorporates the degrees of the proteins in that there are no other precepts used in the sampling, or determination, of the interactions. Therefore, we must conclude that there are other factors involved in the assemblies of the second group of networks. These factors may be artificial or biological. With regard to the former, it is known that there are very small interaction overlaps between HT experimentally determined networks for the same species [13],[59]. While each individual HT network may be representative of the DCDW model, a combined set will not be and, hence, will appear multi-modular. Alternatively, the manual curation of a PPI network may involve a search, or verification, of interaction partners for proteins already present in the intermediate network. Such a process may unintentionally introduce preferential attachments. In the event that a PPI network is not representative of the DCDW model, and any artificial influences can be discounted, then there must be biological actions leading to preferential, or selective, interactions.

### Analysis of Degree Distributions of Protein–Protein Interaction Networks

If PPI networks from a single HT experiment incorporate a significant random element, as indicated above, then it is aberrant that they do not have degree distributions that are Poissonian in nature. Rather, HT experiments consistently generate PPI networks that have degree distributions that resemble power-law scaling. Therefore, they must contain some elements that distinguish them from ER random graphs. The findings described above suggest that the organizations of these PPI networks may be dependent only upon their degree distributions; i.e., it is the protein degrees that determine the observed interactions, rather than the converse. Such an interpretation would imply that the HT experiments are observing the ability to bind, rather than specific interactions that occur in the cell. If so, it should be possible to evolve degree distributions of PPI networks without the use of a network framework, i.e., we wish to model the evolution of the proteins' “binding affinities.”

**Figure 6:**
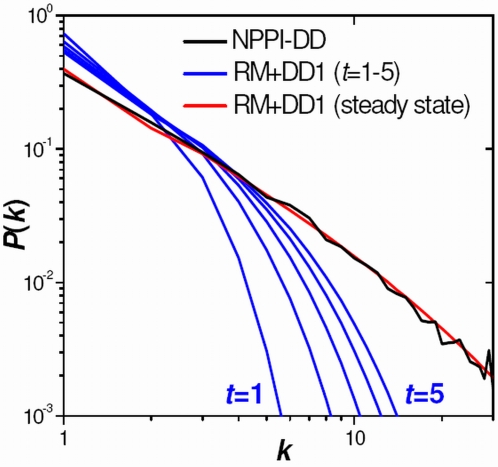
Degree Distributions Representative of PPI Networks and of the RM+DD1 Model. The normal PPI degree distribution (NPPI-DD) (black) is determined by averaging over eight PPI networks. Degree distributions of the RM+DD1 model are shown at time steps 1–5 (blue) and the steady state (red).

There are well known network models that are able to generate graphs with power-law-type scaling degree distributions. These are based on a number of concepts, including preferential attachment [33],[34], duplication [35]–[37], and hierarchical [38],[39] approaches. However, these have typically not been shown to reproduce degree distributions of actual PPI networks. We use a degree distribution averaged over the eight individual HT datasets (listed in the top group of Table 1) as a template for PPI networks. This degree distribution, illustrated in black in Figure 6, is subsequently referred to as the normal PPI degree distribution (NPPI-DD). The NPPI-DD is not shown for degrees higher than 30 since this region includes more noise. It is clear that, overall, the NPPI-DD resembles power-law scaling; however, this scaling is somewhat more level in the low-degree region. This type of deviation from perfect power-law scaling has been noted previously for PPI networks [32].

Our model of protein degree evolution initializes by setting all protein degrees to be equal to one, i.e., the degree distribution at time *t* = 0 is represented by *P_t_*_ = 0_(*k* = 1) = 1, where *P_t_*(*k*) represents the fraction of proteins having a degree *k* after time step *t*. During the first phase of the next time step, we postulate that all protein degrees are able to randomly mutate, and we model the total effect of the mutations into the degree distribution by use of the Poisson distribution:
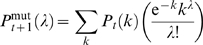
(2)Here, 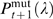 is the resultant degree distribution from the random mutation phase, and the term in braces is analogous to that seen in Equation (1), i.e., the probability that a protein of initial degree *k* will become degree *λ*. For *t* = 0, the summation reduces to one term, but for latter time steps the degree distribution is more diverse. We note that this procedure will result in some proteins having zero degree. During the second phase of this time step, after the mutation phase, we postulate that all proteins of degree one will duplicate. The reasons for duplicating only proteins of single degree are discussed below. This duplication phase is mathematically represented by:
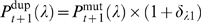
(3)where *δλ*_1_ is the Dirac delta function and is equal to one if *λ* = 1 and is zero otherwise. The final degree distribution at the end of the time step is obtained by discarding proteins with zero degree and renormalizing:
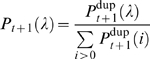
(4)There are two reasons for eliminating proteins of zero degree: (i) all proteins of degree zero will remain degree zero in subsequent time steps, and (ii) most experiments do not report the proteins that have no observed interactions.

The two-step procedure described above, random mutation followed by duplication of degree-one proteins (RM+DD1), can be iterated over a number of time steps by cycling through Equations 2–4. Figure 6 shows RM+DD1 degree distributions for time steps *t* = 1 through *t* = 5 as blue lines. At *t* = 0 the distribution is simply *P*_0_(1) = 1. The curves clearly show that the degree distribution decays less abruptly with additional time steps and approaches some converged profile. Our computations indicate that the degree distribution is essentially converged after 100 time steps; therefore, the evolved distribution following a billion steps, shown in red in Figure 6, is representative of the steady state. This steady-state distribution, for the RM+DD1 model, is seen to almost exactly overlap the NPPI-DD.

The near-perfect agreement between the RM+DD1 steady-state and the NPPI-DD may be coincidental; however, an analysis of its foundations is warranted. Firstly, we are modeling only the evolution of protein degrees, i.e., the number of binding partners a protein has. There is no attempt to describe any network of interactions or any specific sub-network of protein interactions describing a particular biological function. The degree strictly represents the ability of proteins to bind, regardless of whether such an interaction is actually utilized in a biological system. Secondly, the justification for the random mutation phase is straightforward and is manifested in the long history of gene mutation research [60]. Here, the mutation concept is applied directly to the degree of a protein rather than to its sequence. We surmise that changes in sequence coincide with changes in behaviors, and the latter includes the degree. Lastly, we include a duplication, or growth, phase that is well substantiated from Ohno's hypothesis on genome growth by duplication [61] and more recent genomic studies [62]. There are two ways to justify the doubling of the degree-one phase: (i) proteins with degree one are purported to evolve faster [58],[63] and (ii) “new” proteins are likely to have a small number of interacting partners. However, there is no strict justification for duplicating proteins of only degree one. There is, obviously, a mathematically infinite number of ways to grow the number of proteins of each degree. Nonetheless, it is curious that exact duplication of only degree-one proteins yields a steady-state degree distribution nearly identical to the NPPI-DD. Whether or not there is biological justification for this element requires further investigation.

There are other non-network-based schemes [64]–[66] that generate property distributions, such as flicker noise or fitness of species, which converge to a critical point that resembles power-law scaling. However, these approaches rely upon the specifications of barrier thresholds that govern whether a spill over, or catastrophic event, occurs. Our model requires no such parameters. In fact, besides defining the mode of growth, the model is based on completely random events modeled by the Poisson distribution. This aspect complements the apparent random natures, discerned above, of the single-experiment determined PPI networks and supports the interpretation that these experiments may be witnessing the ability to bind.

## Discussion

Here we have expanded on a previous study that demonstrated that the interactions in PPI networks incorporate DW elements, i.e., that the probability of an interaction between two proteins is generally proportional to the product of their degrees [54]. This finding prompted the employment of a network model that constructs a DW network, while preserving an input degree distribution. This DCDW approach can be considered similar to a previously reported random-type graph model [44] in that a comparable construction procedure is used, however, a subtle difference is that the DCDW model maintains consistent nodal weights, equal to their input degrees, throughout the construction procedure. The DCDW model was shown to exactly reproduce properties of ER random graphs, when provided with degree distributions for the latter, and therefore we utilize it as a random network model. This DCDW model is shown to closely reproduce the topological properties of eight PPI networks, each assembled from an individual HT experiment. Furthermore, the PPI networks and the DCDW model were shown to contain similar clustering and path length profiles, which illuminated the relationships with degree. The results lend further support to the premise that DW behavior is intrinsic to these PPI networks and, therefore, indicative of a significant random element. Thus, it is reasonable to conclude that the connectivities in these PPI networks have substantial random characteristics for the observed degree distributions. We are not implying that the experiments are generating random interactions, rather we perceive that the interactions have evolved using a random-influenced mechanism over time and that the experiments may be observing the ability of proteins to bind. While Y2H data are known to be noisy, the two PPI networks of *E. coli* have been determined using a different methodology (TAP), yet each also has a close similarity to the DCDW model. Consequently, these findings may be relevant to any HT technology.

The apparent inclusion of randomness in the individual HT PPI networks precipitated the development of a model to describe the evolution of protein degrees, or binding affinities. We show that by initializing all nodes to have a single interacting partner and iteratively applying mutation and growth modulations (of degree-one nodes), a steady-state degree distribution that resembles a power law results. Moreover, this steady-state degree distribution is found to be almost identical to a degree distribution computed by averaging over eight HT experimental PPI networks. Therefore, we postulate that the resemblance of the observed PPI degree distributions to power-law scaling is simply a result of growth and random mutation over time. This type of evolution mechanism is not surprising, but the exceptional agreement suggests that we are capturing the essence of the process. The model is consistent with an evolutionary process driven by single gene duplications followed by slow continuous genetic drift of all proteins. This interpretation is compatible with the following observations on PPI networks: (i) essential proteins typically have high degrees [29]. From the evolution of the network, the fundamental genes that can sustain life appeared first. As they evolved via gene duplication and mutation, they acquired more degrees. Hence, they may now be found among the highest degree nodes. (ii) The evolutionary rate of a protein correlates inversely with its degree [63],[67]. Proteins with a greater number of interactions are more likely to have existed longer and, therefore, more likely to have incorporated additional mutations. As such, their rates of change may have slowed, being closer to a steady state. We anticipate that the mutation and growth model can be generalized and applied to other types of evolving real-world systems to provide qualitative and quantitative simulations.

In contrast to the HT PPI networks, the curated and high-confidence PPI networks have global properties that vary significantly from the DCDW model. These differences can be attributed to two main reasons. Firstly, if a curated network includes interactions from more than one HT data set, and the overlap between these sets is very small, then the curated network may be essentially multi-modular. While the individual HT data sets may be well represented by the DCDW model, the combined network may not be due to unintentionally introduced partiality in the interactions. An exception exists if the HT datasets are highly complementary and the merged set is representative of a single DW module. Secondly, curated and high-confidence PPI networks have been manually manipulated and, therefore, include biases, or preferential influences, upon the protein interactions which may or may not be representative of the underlying biology. For these networks, the DCDW model will not be an accurate representation as it includes no such constraints.

An important consideration is that HT methods may generate many false-positive interactions. If these false positives far outnumber the true, or real, interactions, then the total PPI network will appear systematically biased depending upon the mode of generation of the false positives. If so, the DCDW model is mimicking this bias rather than the true biology and, therefore, provides clues as to the origin of the false-positive interactions.

Many studies infer biological properties, or traits, by contrasting PPI networks against corresponding NULL networks, which are akin to DCDW networks. The PPI networks used are often downloaded from databases that have curated interactions from a number of sources, including HT experiments. While the individual HT datasets will have affinities to their corresponding NULL networks, as demonstrated in this work, the curated datasets will not. Therefore, any conclusions or inferences drawn from these studies should be treated with caution. The elucidation of guiding principles in biology is frequently contingent upon contrasts to randomness. However, care must be taken to ensure that the data are not artificially modulated, as in the case of many curated PPI networks.

The findings reported here indicate that HT PPI networks incorporate random interactions between proteins of varying binding affinities. The evolution of the proteins' affinities can be modeled by a mechanism based upon duplication and random mutation for which the steady degree distribution is almost identical to one averaged over eight HT experimental PPI networks. However, curated and high-confidence PPI networks are found to contain influences exogenous to the HT experiments, leading to preferential associations between protein pairs. These results provide a means to distinguish uninhibited network organization with respect to the observed degree distribution and may shed light for the identification of consistent influences leading to preferentially connected networks representing manual curation and/or the underlying biology.

## Supporting Information

Text S1. Mathematical Relationship between Randomness and Degree-Weighted Behavior
(0.02 MB PDF)

Figure S1. Dependence of Clustering Coefficient upon Node Degree for Four PPI Networks and Their DCDW Equivalents. (A) *Caenorhabditis elegans*, (B) *Saccharomyces cerevisiae* (*Y2H*), (C) *Helicobacter pylori*, and (D) *Plasmodium falciparum*. Clustering profiles for the PPI networks (black) and the corresponding tenth (blue) and fiftieth (red) realizations of the DCDW model.
(0.01 MB PDF)

Figure S2. Dependence of Path Length upon Node Degree for Four PPI Networks and their DCDW Equivalents. (A) *Caenorhabditis elegans*, (B) *Saccharomyces cerevisiae* (*Y2H*), (C) *Helicobacter pylori*, and (D) *Plasmodium falciparum*. Path length profiles for the PPI networks (black) and the corresponding tenth (blue) and fiftieth (red) realizations of the DCDW model.
(0.01 MB PDF)

Video S1 Three-Dimensional Animation of the *Escherichia coli* (HT2) PPI Network of Arifuzzaman et al. [13] A total of 3047 proteins and 11477 interactions are shown. The top 20 most connected proteins (hubs) and the interactions between them appear in red. All other proteins and interactions appear in translucent grey.
(18.37 MB AVI)
