# Supplementary material for: Probing the Extent of Randomness in Protein Interaction Networks
Source: PLoS Comput Biol. 2008 Jul 11;4(7):e1000114. doi: 10.1371/journal.pcbi.1000114 (PMC2527968; doi:10.1371/journal.pcbi.1000114)
Supplement: Text S1. — Mathematical Relationship between Randomness and Degree-Weighted Behavior (0.02 MB PDF) [file pcbi.1000114.s001.pdf]

Mathematical Relationship between Randomness and Degree-Weighted Behavior

Suppose that in a random network containing M nodes, two of the nodes, i and j, have degrees k_i and k_j, respectively. In this case the number of ways that they can interact with other nodes, *but not with each other*, is given by:

$$Q_{i \times j} = \binom{M-2}{k_i}\binom{M-2}{k_j}.$$

Alternatively, *if nodes i and j interact (once)*, then in this special case the number of ways that they can interact with the other nodes is given by:

$$Q_{i-j} = \binom{M-2}{k_i - 1}\binom{M-2}{k_j - 1}.$$

Therefore, the probability that nodes i and j will have an edge is given by:

$$P_{ij} = \frac{Q_{i-j}}{(Q_{i \times j} + Q_{i-j})} = \frac{k_i k_j}{(M - k_i - 1)(M - k_j - 1) + k_i k_j}.$$

For the usual case when M is much larger than the degrees k_i and k_j, this implies that $P_{ij} \propto k_i k_j$. Therefore, the overwhelming majority of interactions in a random network will typify degree-weighted (DW) behavior. The only interactions that will deviate slightly from DW behavior will be between nodes having extremely large degrees. However, in such a case, the probability of an edge between these nodes will be very slightly increased, if at all noticeable. We understand that the analysis above does not take into account the limitations in the degrees of nodes other than i and j. However, we do not anticipate that inclusion of these constraints will significantly alter the conclusions.
