# Supplementary figures and images for: Probing the Extent of Randomness in Protein Interaction Networks
Source: PLoS Comput Biol. 2008 Jul 11;4(7):e1000114. doi: 10.1371/journal.pcbi.1000114 (PMC2527968; doi:10.1371/journal.pcbi.1000114)

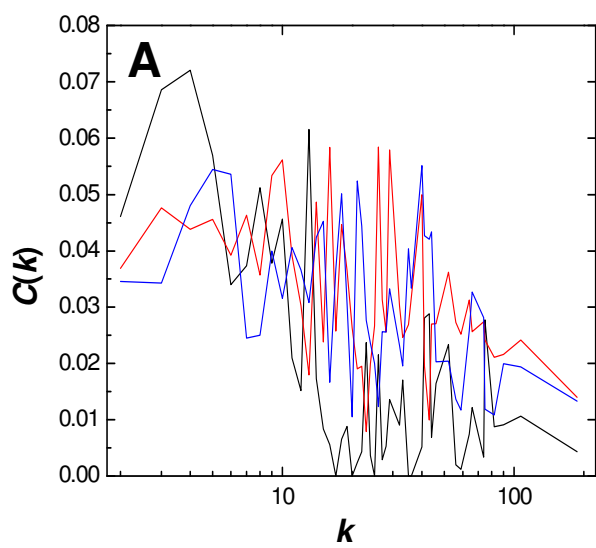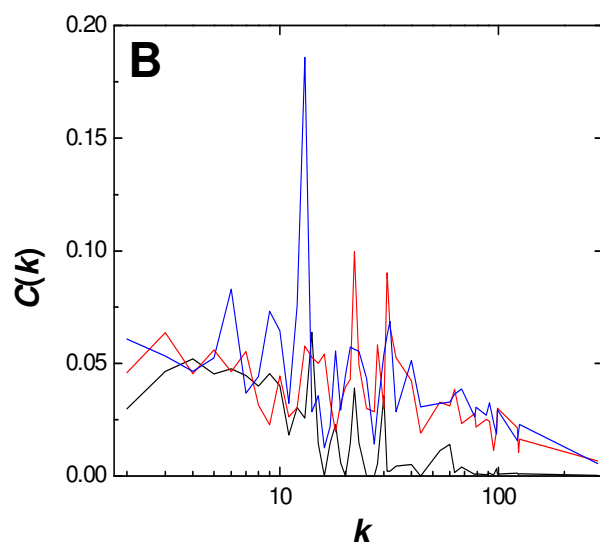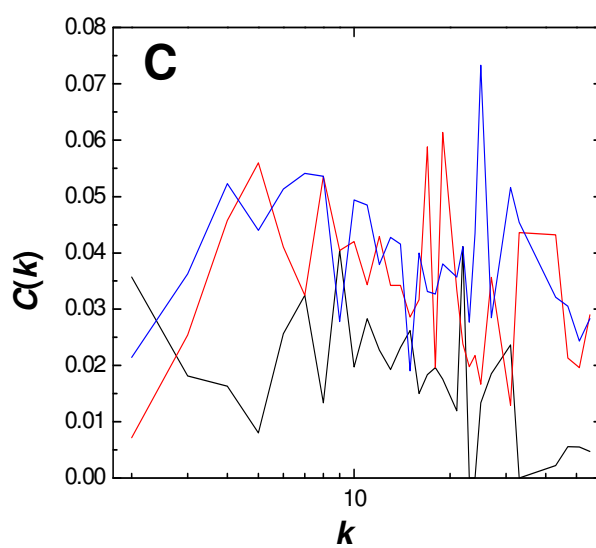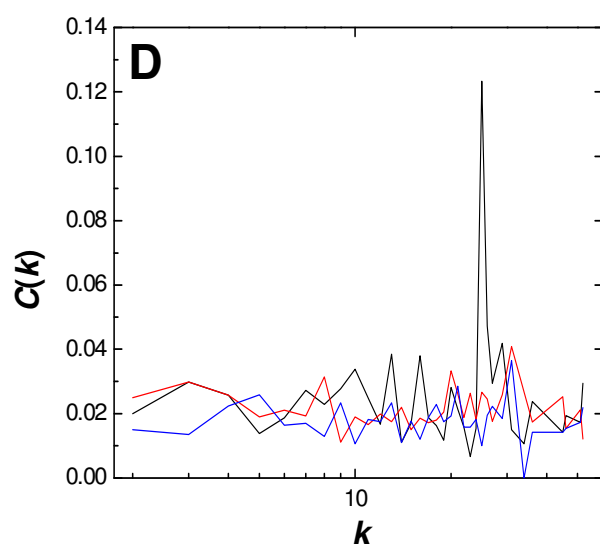

**Figure S1**

Supplement: Figure S1. — Dependence of Clustering Coefficient upon Node Degree for Four PPI Networks and Their DCDW Equivalents. (A) Caenorhabditis elegans, (B) Saccharomyces cerevisiae (Y2H), (C) Helicobacter pylori, and (D) Plasmodium falciparum. Clustering profiles for the PPI networks (black) and the corresponding tenth (blue) and fiftieth (red) realizations of the DCDW model. (0.01 MB PDF) [file pcbi.1000114.s002.pdf]

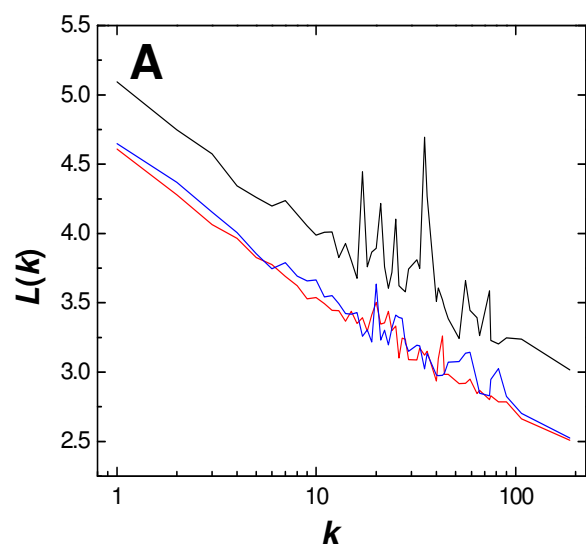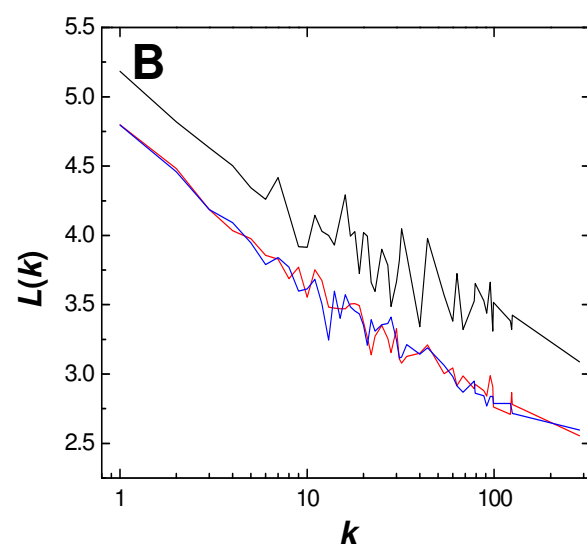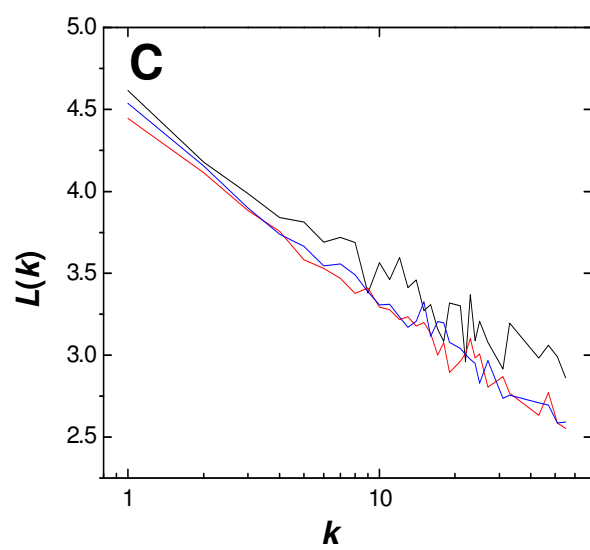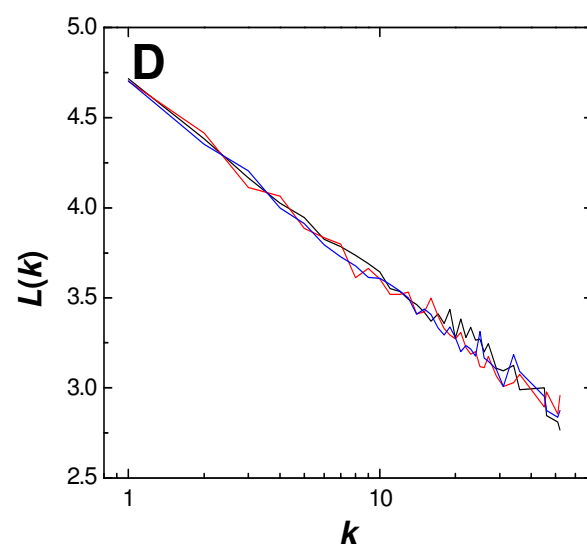

**Figure S2**

Supplement: Figure S2. — Dependence of Path Length upon Node Degree for Four PPI Networks and their DCDW Equivalents. (A) Caenorhabditis elegans, (B) Saccharomyces cerevisiae (Y2H), (C) Helicobacter pylori, and (D) Plasmodium falciparum. Path length profiles for the PPI networks (black) and the corresponding tenth (blue) and fiftieth (red) realizations of the DCDW model. (0.01 MB PDF) [file pcbi.1000114.s003.pdf]
